# Caesarean section rate and cost control effectiveness of case payment reform in the new cooperative medical scheme for delivery: evidence from Xi County, China

**DOI:** 10.1186/s12884-018-1698-0

**Published:** 2018-03-09

**Authors:** Shuang Liu, Jing Wang, Liang Zhang, Xiang Zhang

**Affiliations:** 1 Department of Health Management, School of Medicine and Health Management, Tongji Medical College, Huazhong University of Science and Technology, Wuhan, Hubei 430030 China; 2 The Key Research Institute of Humanities and Social Science of Hubei Province, Huazhong University of Science and Technology, Wuhan, Hubei 430030 China

**Keywords:** Cost control, Integrated case payment, New cooperative medical scheme, Caesarean section rate, Delivery

## Abstract

**Background:**

In China, increases in both the caesarean section (CS) rates and delivery costs have raised questions regarding the reform of the medical insurance payment system. Case payment is useful for regulating the behaviour of health providers and for controlling the CS rates and excessive increases in medical expenses. New Cooperative Medical Scheme (NCMS) agencies in Xi County in Henan Province piloted a case payment reform (CPR) in delivery for inpatients. We aimed to observe the changes in the CS rates, compare the changes in delivery-related variables, and identify variables related to delivery costs before and after the CPR in Xi County.

**Methods:**

Overall, 28,314 cases were selected from the Xi County NCMS agency from 2009 to 2010 and from 2014 to 2015. One-way ANOVA and chi-square tests were used to compare the distributions of CS and vaginal delivery (VD) before and after the CPR under different indicators. We applied multivariate linear regressions for the total medical cost of the VD and CS groups and total samples to identify the relationships between medical expenses and variables.

**Results:**

The CS rates in Xi County increased from 26.1% to 32.5% after the CPR. The length of stay (LOS), total medical cost, and proportion of county hospitals increased in the CS and VD groups after the CPR, which had significant differences. The total medical cost in the CS and VD groups as well as the total samples was significantly influenced by inpatient age, LOS, and hospital type, and had a significant correlation with the CPR in the VD group and the total samples.

**Conclusion:**

The CPR might fail to control the growth of unreasonable medical expenses and regulate the behaviour of providers, which possibly resulted from the unreasonable compensation standard of case payments, prolonged LOS, and the increasing proportion of county hospitals. The NCMS should modify the case payment standard of delivery to inhibit providers’ motivation to render CS services. The LOS should be controlled by implementing clinical guidelines, and a reference system should be established to guide patients in choosing reasonable hospitals.

## Background

### Caesarean delivery and its risk

The global rise in caesarean section (CS) rates has resulted in considerable concern. In 1985, the World Health Organization (WHO) first recorded the “highest” CS rate of 15% [1]. However, CS rates remain higher than what is considered reasonable in most areas [2]. In 2016, according to data collected from all continents, the highest CS rates were 40.5% for Latin America and the Caribbean region; these rates were above 15% for all other regions, except for Africa, which has a CS rate of 7.3% [3].

China has experienced remarkable increases in the number of CS procedures, and the CS rate has grown by more than 30% since 1992 [4]. Before the 1990s, the CS rate was below 10% because the development of modern medicine in China was in the early stages. However, this rate rose rapidly. Between 1988 and 2008, the CS rate increased from 3 to 39%, which initially occurred in urban areas and then later in the rural areas with the improvement of the health system [5]. The rapid increase in CS has evoked concern about the health system in China. Therefore, a series of measures have been taken to reduce the unreasonable CS rates, including the reformation of the payment system, the normalization of clinical guidelines, and the strengthening of health education. Accordingly, the growth of the CS rate has slowed and reached 34.9% in 2014 [6].

High CS rates have led to a series of problems. CS is a costly operation compared to vaginal delivery (VD); consequently, it could aggravate the financial burden for households and health care systems and lead to unnecessary risks for maternal and neonatal health [7, 8]. Because CS are subsidized to different degrees, the increased use of CS in many developing countries has resulted in an increase in government funding and a substantially higher proportion of government funding in the total health-care expenditures [9]. From a global perspective, excess use of CS has had considerable costs [10]. Additionally, CS has been linked to a risk of surgical site infections, as described by Eriksen et al. [11], and CS increases the risk of early childhood anaemia compared with VD, as confirmed by Li et al. [12].

Although many objective factors led to the CS rate increase, such as the obstetric care system, provider factors, and pregnant factors, it is likely that social factors, not medical factors, are the main cause for the rate increase in China [13]. These non-medical reasons for the high proportion of CS could be motivated by the preferences of pregnant women and providers. In the absence of medical indications, many pregnant women who insist on requiring CS generally mistakenly believe that CS is less painful and more convenient than VD [14]. Physicians often favour the rapid surgical turnover over a potentially long labour and have incentives to perform CS because they fear charges of malpractice [15]. High medical insurance coverage could also be a key reason for the high CS rates among both providers and patients. Bogg et al. [16] found that the unhealthy increases in CS rates in rural China resulted from the design of the NCMS, the payment systems, and revenue-related bonus systems for doctors. Hu et al. [17] previously emphasised that providers and patients were less likely to avoid excessive tests and medication when a large proportion of the costs were shifted to third-party payers, which leads to increased medical expenses.

To reduce the CS rates and control delivery costs, many countries have adopted various measures and methods, which can be roughly classified into 3 strategies: (1) health education and teaching correct delivery concepts in society, (2) payment reform, and (3) medical malpractice reform [18]. China aims to modify and improve the medical insurance payment system in many places to reduce the CS rates and control delivery costs.

### Medical insurance in China and payment reform in Xi County

Through the end of 2016, the health insurance system in China has had three main parts: (1) the Urban Employee Basic Medical Insurance Scheme, (2) the Urban Resident Basic Medical Insurance Scheme, and (3) the NCMS. The NCMS, which was established in 2003, aims to cover the medical expenses of rural residents through financial assistance from the central government, local government, and rural residents themselves, with a separate pool of funds for each county. The annual premium per capita of the NCMS was raised from 30 Chinese Yuan (¥) (USD 4.35, according to the exchange rate of USD 1 = ¥6.89 in April 1, 2017) in 2003, of which ¥20 (USD 2.90) came s from the government and ¥10 (USD 1.45) came from the rural residents, to an average of ¥410(USD 59.50) in 2014 and ¥570 (USD 82.72) in 2016, of which ¥420 (USD 60.95) came from the government and ¥150 (USD 21.77) came from the rural residents. Until 2015, the NCMS covered 99% of all rural residents. However, in 2017, the NCMS began gradually being integrated into the Urban Resident Basic Medical Insurance scheme.

To control the increase in medical expenses, the NCMS agencies in some counties have attempted to implement payment reform for providers by transforming the fee-for-service (FFS) payment into case payment. Because of the FFS payment, which is the most popular method of paying providers in the NCMS, incentivizes health providers to generate unnecessary procedures for high profits and resulted in the inefficient utilization of health services, it has played an important role in the increasing medical expenses in China [19–21]. Therefore, case payment, which is a prospective payment system (PPS), is considered a useful measure for regulating the behaviour of health providers and ultimately controlling the unreasonable increases in medical expenses. Both experts and governors consider the use of case payment more suitable than the use of diagnosis-related groups in rural China because of the limitation of the medical information system and providers’ ability to code procedures. McCue et al. [22] testified that the case payment approach reduces costs by approximately 6%–10% compared with FFS arrangements.

Accordingly, the NCMS agencies in some counties, such as Xi County, Henan Province, China, have explored the use of case payment through some support policies to control the increase in medical expenses. In Xi County, which is predominantly occupied by rural residents who are covered by the NCMS, a pilot CPR for inpatients in the NCMS began in 2009 and was implemented for the entire county in 2011. Delivery was the first case covered by the CPR in Xi County. The effectiveness of the CPR to control costs in Xi County requires further examination.

To provide insight into the reform and determine its value as a model for other districts that intend to implement such policies, we take delivery as a research object and investigated the effectiveness of the CPR on CS rate reduction and delivery cost control. In the present study, we aim to (1) observe the changes in the CS rates and delivery costs before and after the CPR, (2) compare the changes in delivery-related variables between CS and VD before and after the CPR, and (3) identify variables related to delivery costs in Xi County.

## Methods

### Data collection

Data were obtained from the inpatient information database of the Xi County NCMS agency. This database contains the claim information of individuals residing in Xi County, which was covered by the NCMS from January 1, 2009, to December 31, 2010, and from January 1, 2014, to December 31, 2015. In total, 34,210 admissions for delivery were found among a total 295,796 inpatient admission cases over the past four years. In China, township hospitals are grass-root institutions, only providing basic medical services and treating. County hospitals are higher level institutions than township hospital in the county. County hospital could provide comprehensive medical services and treat both ordinary delivery without complication and severe case with complication, because their medical service capacity is stronger than township hospitals.

To focus on studying the effectiveness of the CPR, we excluded patients who were not covered by the case payment for unknown reasons in the hospital, patients for whom the delivery method (VD vs. CS) could not be determined, and patients who visited from outside Xi County. A total of 28,314 deliveries were analysed.

We considered hospital deliveries from January 1, 2009, to December 31, 2010, as the pre-CPR patients and deliveries from January 1, 2014, to December 31, 2015, as the post-CPR patients. There were 12,705 deliveries in the pre-CPR group and 15,609 in the post-CPR group.

### Statistical analysis

To ensure the comparability of the medical costs of delivery, we adjusted the medical costs using the consumer price index (2010, 2014, and 2015 = 102.8, 101.8, and 101.1, respectively) in Henan Province.

The variables in this study included inpatient age, LOS, total medical cost, CPR, hospital type, and delivery type. The inpatient age, LOS, and total medical cost were the continuous variables, whereas CPR, hospital type and delivery type were the categorical variables. Total medical cost refers to the full costs of hospitalization. CPR includes the pre-CPR and the post-CPR, and there were two types of hospitals: township and county hospitals. Two types of delivery: VD and CS.

We compared the variables of the VD and CS groups between the pre-CPR and post-CPR, respectively. The variables include the inpatient age, LOS, total medical cost and hospital type. The continuous variables were analysed using a one-way ANOVA to compare the differences in the means, and the categorical variables were analysed using a chi-squared test to compare the differences in proportions.

We applied multivariate linear regressions for the total medical cost of the VD and CS groups and total samples to identify the relationship between medical expenses and variables. The variables include age, CPR, LOS, hospital type and delivery type. Delivery type was only taken into Model 3 (total samples). CPR, hospital type and delivery type are dichotomy variables. Dummy variables can quantify the dichotomy variables and be incorporated in regression models [23]. In order to set reference variables for these three dichotomy variables in multivariate linear regressions, we set (1. the pre-CPR = 0 versus the post-CPR = 1, (2. township hospital = 0 versus county hospitals = 1, and (3. VD = 0 versus and CS = 1, respectively [24]. All data were analysed using SPSS 19.0. All *P* values used in these tests were two-tailed, with 0.05 indicating statistical significance.

## Results

Table 1 shows the CS rate of Xi County before and after the CPR. The number of cases in the VD and CS groups increased after the CPR, but there was a statistically significant increase in the CS rates of rural women in Xi County from 26.1% before the CPR to 32.5% after the CPR.

**Table 1:** Cesarean rate of Xi County before and after CPR

Mode of delivery	pre-CPR	post-CPR	total	% change in CS rate	*p*-value
Total	12,705	15,609	28,314		
CS (N (%))	3319 (26.124)	5069 (32.475)	8388	+ 6.351%	<0.001
VD (N (%))	9386 (73.876)	10,540 (67.525)	19,926		

Table 2 presents the variable differences between the pre-CPR and post-CPR of the CS and VD groups, respectively. All the variables, except age, showed significant differences in the CS and VD groups between the pre-CPR and post-CPR, respectively. The LOS of the post-CPR is longer than that of the pre-CPR in each group, especially for the VD group, which increased from 2.79 days to 4.59 days. Similarly, the total medical cost post-CPR was higher than pre-CPR for each group. Another phenomenon that should be noticed is that the proportion of county hospitals increased markedly from 70.50% to 95.86% in the CS group and 71.26% to 80.89% in the VD group after the CPR.

**Table 2:** Descriptive variables comparison between pre-CPR and post-CPR of Xi County

	pre-CPR		post-CPR		*p*-value ((1) VS (3))	*p*-value ((2) VS (4)
	CS (1) (*N* = 3319)	VD (2) (*N* = 9386)	CS (3) (*N* = 5069)	VD (4) (*N* = 10,540)		
Age (years) (mean(SD))	25.92 (5.577)	25.50 (5.194)	25.99 (4.738)	25.598 (4.324)	0.551	0.142
LOS (days) (mean/SD)	6.55 (6.737)	2.786 (6.784)	6.94 (5.099)	4.590 (7.675)	0.004	<0.001
Total medical cost (USD) (mean/SD)	502.12 (950.967)	170.32 (643.585)	558.90 (507.763)	242.31 (514.039)	<0.001	<0.001
Type of hospital					<0.001	<0.001
Township Hospital (*N* (%))	979 (29.500)	2716 (28.936)	210 (4.142)	2014 (19.108)		
County Hospital (*N* (%))	2340 (70.500)	6670 (71.063)	4859 (95.856)	8526 (80.892)		

*α = 0.05

Table 3 presents the results of the multivariate linear regression analysis to explain the relationships among the variables and the total medical costs of the CS and VD groups and the total sample. In the CS group (Model 1), almost all variables, including inpatient age, LOS, and hospital type but not the CPR, were significantly correlated with the total medical cost. Additionally, these three factors are risk factors that affect the total medical cost. Furthermore, in the VD group (Model 2), all variables were significantly related to the total medical cost. The LOS and hospital type are also risk factors for the total medical cost. The CPR is also a risk factor. However, age becomes is a protective factor for the total medical costs. In the total samples (Model 3), all variables have a relationship with the total medical cost; CPR, LOS, hospital type, and delivery type are risk factors, but age is a protection factor. Accordingly, the total medical cost among the three regression models were significantly influenced by inpatient age, LOS, and hospital type, but the LOS and hospital type were the risk factors in each model, whereas age was a protection factor for the VD group and total samples. In addition, the total medical cost in the CS group had no relation to the CPR, despite showing a significant correlation with costs in the VD group and the total samples. The higher the level of the delivery was in hospitals, the longer the LOS was and the higher the total medical cost was.

**Table 3:** Multivariate linear regressions on total medical cost in Xi County

	Factor		B	S.E.^a^	Beta^b^	t	*p*-value^b^
Model 1 (CS)	Constant		578.684	41.200		14.046	< 0.001
	Age		3.496	1.049	0.024	3.332	< 0.001
	CPR	pre-CPR^c^					
		post-CPR	−21.090	11.715	−0.014	−1.800	0.072
	LOS		4.924	0.929	0.039	5.298	< 0.001
	Hospital type	Township hospital^c^					
		County hospital	1617.581	16.538	0.761	97.811	< 0.001
	R^2^		0.566				
Model 2 (VD)	Constant		− 215.343	22.027		−9.776	< 0.001
	Age		−5.800	0.652	−0.044	−8.895	< 0.001
	CPR	pre-CPR^c^					
		post-CPR	405.942	6.309	0.322	64.343	< 0.001
	LOS		1.446	0.432	0.017	3.349	< 0.001
	Hospital type	Township hospital^c^					
		County hospital	895.982	7.431	0.606	120.567	< 0.001
	R^2^		0.518				
Model 3 (total)	Constant		−493.046	20.014		−24.635	< 0.001
	Age		−2.474	0.577	−0.010	−4.289	< 0.001
	CPR	pre-CPR^c^					
		post-CPR	315.185	5.779	0.128	54.540	< 0.001
	LOS		2.610	0.413	0.015	6.325	< 0.001
	Hospital type	Township hospital^c^					
		County hospital	1030.098	7.131	0.341	144.457	< 0.001
	Delivery type	VD^c^					
		CS	2130.934	6.317	0.793	337.314	< 0.001
	R^2^		0.853				

^a^S.E. = Standard Error

^b^α = 0.05

^c^Reference group of categorical predictor variables in multivariate linear regressions

## Discussion

The CS rates in Xi County remained high, and the total medical costs of delivery increased, even after the implementation of the CPR in 2011. Furthermore, the medical costs of delivery increased in the CS and VD groups after adjusting the price inflation by using the consumer price index. The proportion of county hospitals in the CS and VD groups increased markedly after the CPR. Therefore, CPR failed to achieve its objective of controlling the growth of excessive medical expenses and regulating the behaviour of providers.

The consistently high CS rates in Xi County could be related to the unreasonable case compensation standards for CS and VD. The case compensation standard for CS is substantially higher than that of VD. For example, the standard total medical cost of VD and CS were set at ¥1360 (USD 197.39) and ¥3400(USD 493.47), respectively, for a delivery without complications. Therefore, the providers prefer CS for economic incentives [5]. McCue et al. [22] testified that the case payment reduces costs by approximately 6%–10% compared with the FFS arrangements, although this scenario may require many years of research to elicit determine whether there is an apparent effect on costs, depending on how high the payment levels were initially set [25, 26]. However, the gap between the reimbursement standards of CS and VD may not be sufficiently narrow to eliminate the economic incentives for doctors, and doctors may still be prompted to choose CS instead of VD. The CPR cannot solve the problem of service regulation when the compensation standards are not designed reasonably, even if the payment reform could affect the service choice of the providers. Controlling the CS rate requires strict clinical indications and guidelines to serve as regulatory tools. Althabe et al. confirmed that clinical guidelines and the policy of a mandatory second opinion decreased the CS rate by 25%, showing that clinical guidelines have a clear effect on reducing the CS rate [27].

The increasing total medical cost of delivery could be a result of not only the increase of the CS rate in Xi County but also the unreasonable compensation standards for case payment, the prolonged LOS, and the increasing proportion of county hospitals. Besides the fact that the case compensation standard of CS was higher than that of VD, the case compensation of CS and VD in Xi County was set to be higher than the average level from the past years, promoting the involvement of a substantial number of hospital and health providers in the CPR. For example, the standard total medical cost of VD was set to ¥1360 (USD 197.39) for delivery without complication and ¥1632 (USD 236.87) for delivery with complications, which were both higher than the real average total medical cost of VD (¥1297.94, USD 188.38) before the CPR. The standard total medical cost of CS was set to ¥3400 (USD 493.47) for delivery without complication and ¥4080 (USD 592.16) for delivery with complication, but the average total medical cost of CS before the CPR was ¥3304.57 (USD 479.62). Consequently, the average medical cost of delivery was substantially higher after the CPR than it was before the CPR.

The prolonged LOS was another reason for the total medical cost increase because the LOS is proportional to the total medical cost [28]. Taheri et al. emphasised that the LOS is a mature indicator of hospital cost reduction and that reducing LOS by 1 day reduces the overall cost of care by an average of 3% or less, which demonstrates a direct link between the LOS and cost [29]. The LOS in CS and VD patients in Xi County increased after the CPR to 6.94 and 4.59 days, respectively. Additionally, the change of the LOS in VD is more significant compared to CS. However, in the United States, the LOS for CS and VD was only 3.01 and 1.74 days, respectively [30]. Generally, case payment can shorten the LOS, but the Xi County case payment extended the LOS, possibly because the LOS can be affected by the availability or lack of local medical level services, social culture, and postpartum family care [31]. However, in this study, no specific evidence was found to explain the prolonged LOS.

A phenomenon that should not be ignored is that the proportion of county hospitals increased tremendously, which may lead to excessive growth in the total medical cost because the cost standards of county hospitals are higher than those of township medical institutions. For example, the standard total medical cost of VD was set to ¥1050 (USD 152.40) for delivery without complication in township hospitals, but in county hospitals, the cost was set to ¥2400 (USD 348.33). Additionally, the standard total medical cost of CS without complications was set to ¥2800 (USD 406.39) in township hospitals and was set to ¥3400 (USD 493.47) in county hospitals. Therefore, the total medical cost of CS and VD increased with an increase in the delivery hospital level. In China, a study verified that the higher the hospital’s level, the more it might cost, which could be explained by the fact that high-level hospitals are much more likely to use expensive drugs and diagnostic tests [32].

Our study has several limitations. First, we used claim data, which lacked vital information on the severity of the pregnancy and ex-experience of the delivery. Therefore, we could not determine the effects of previous medical history on our results. Furthermore, the limited information regarding the social-economic characteristics of the inpatients resulted in the lack of an extensive analysis of residents to explain the conclusion. Additionally, because there was no control group to compare with Xi County, the effectiveness of the CPR in Xi County was tested using only a before-and-after contrast analysis. It was not confirmed with a difference-in-difference estimation, which is an effective method for reducing bias when comparing groups and can produce considerably accurate and effective estimates [33].

## Conclusion

The purpose of the CPR was to control the unreasonable growth of medical expenses and regulate the behaviour of providers. However, our results confirmed that it failed to achieve its goal because the CS rate remained at a high level and the total medical cost continued to increase. The consistently high level of CS rates in Xi County could be related to the unreasonable case compensation standards of CS. The continuing increase in the total medical cost of delivery could be a result not only of the growth in CS rates in Xi County but also the unreasonable compensation standards for case payment, the prolonged LOS, and the increasing number of county hospitals. Therefore, the NCMS should modify the case payment standard of delivery for CS and VD to inhibit the motivation of providers to offer CS services. The LOS should be strictly controlled by implementing clinical guidelines, and a reference system should be established to guide patients to choose reasonable hospitals.

## Abbreviations

CPR: Case payment reform
CS: Caesarean section
FFS: Fee for service
LOS: Length of stay
NCMS: New Cooperative Medical Scheme
PPS: Prospective payment system
VD: Vaginal delivery
